# BTZO-15, an ARE-Activator, Ameliorates DSS- and TNBS-Induced Colitis in Rats

**DOI:** 10.1371/journal.pone.0023256

**Published:** 2011-08-10

**Authors:** Hiroshi Yukitake, Haruhide Kimura, Hirobumi Suzuki, Yasukazu Tajima, Yoshimi Sato, Toshihiro Imaeda, Masahiro Kajino, Masayuki Takizawa

**Affiliations:** 1 Pharmaceutical Research Division, Takeda Pharmaceutical Company Limited, Fujisawa, Japan; 2 Chemistry, Manufacturing and Controls, Takeda Pharmaceutical Company Limited, Osaka, Japan; Charité-University Medicine Berlin, Germany

## Abstract

Inflammatory bowel disease (IBD) is a group of chronic inflammatory disorders that are primarily represented by ulcerative colitis and Crohn's disease. The etiology of IBD is not well understood; however, oxidative stress is considered a potential etiological and/or triggering factor for IBD. We have recently reported the identification of BTZO-1, an activator of antioxidant response element (ARE)-mediated gene expression, which protects cardiomyocytes from oxidative stress-induced insults. Here we describe the potential of BTZO-15, an active BTZO-1 derivative for ARE-activation with a favorable ADME-Tox profile, for the treatment of IBD. BTZO-15 induced expression of heme oxygenase-1 (HO-1), an ARE-regulated cytoprotective protein, and inhibited NO-induced cell death in IEC-18 cells. Large intestine shortening, rectum weight gain, diarrhea, intestinal bleeding, and an increase in rectal myeloperoxidase (MPO) activity were observed in a dextran sulfate sodium (DSS)-induced colitis rat model. Oral administration of BTZO-15 induced HO-1 expression in the rectum and attenuated DSS-induced changes. Furthermore BTZO-15 reduced the ulcerated area and rectal MPO activity in 2,4,6-trinitrobenzene sulfonic acid (TNBS)-induced colitis rats without affecting rectal TNF-α levels. These results suggest that BTZO-15 is a promising compound for a novel IBD therapeutic drug with ARE activation properties.

## Introduction

Ulcerative colitis (UC) and Crohn's disease (CD) are human inflammatory bowel diseases (IBD) of unknown etiology characterized by chronic relapses of hyperinflammation of the gastrointestinal tract and a dysregulated mucosal immune response [1]. Many factors participate in the pathology of IBD such as oxidative stress, neutrophil infiltration, overproduction of proinflammatory mediators including cytokines, dysfunction of immune systems, and imbalance of microflora [1], [2]. Among these factors, oxidative stress appears to be a major factor in the pathogenesis of IBD and may be the mainstay of disease initiation and perpetuation [3]–[6]. Heme oxygenase-1 (HO-1), an enzyme that degrades heme to carbon monoxide, ferrous iron, and biliverdin, participates in the cellular defense against oxidative stress. Interestingly, up-regulation of HO-1 shows promise as a clinical intervention of IBD [3], [7].

In a previous study, we reported the identification of BTZO-1, 2-pyridin-2-yl-4H-1,3-benzothiazin-4-one, which inhibits the apoptosis of cardiomyocytes by inducing cytoprotective factors, such as glutathione S-transferase (GST) Ya and HO-1 via activation of antioxidant response elements (ARE) [8]. BTZO-2, an active BTZO-1 derivative, protected heart tissues during ischemia/reperfusion injury in rats [8]. BTZO-1 selectively bound macrophage migration inhibitory factor (MIF), and reduction of cellular MIF protein levels by siRNA suppressed BTZO-1-induced GST Ya expression. Therefore, MIF may be a target protein of BTZO-1.

ARE is a cis-acting DNA regulatory element located in the regulatory regions of multiple genes encoding phase II detoxifying enzymes and cytoprotective proteins including GSTs, HO-1, reduced nicotinamide adenine dinucleotide phosphate (NAD(P)H), quinone oxidoreductases (NQOs), UDP-glucuronosyl transferase (UDP-GT), epoxide hydrase, γ-glutamylcystein synthetase (γ-GCS), and peroxiredoxin 1 [9]–[11]. In mammalian cells, activation of the ARE is of critical importance to cellular protection against oxidative stress [10]–[12]. In fact, t-BHQ, which up-regulates a battery of ARE-regulated genes, reportedly protects cells from oxidative damage *in vitro* [13]. There is also a growing body of evidence suggesting that modulation of these cytoprotective genes has profound effects on immune and inflammatory responses. Activation of cytoprotective ARE-regulated genes can suppress inflammatory responses, whereas decreased expression of these genes results in autoimmune disease and enhances inflammatory responses to oxidant insults. For instance, it has been reported that 15-Deoxy-Δ^12, 14^-Prostaglandin J_2_ (15d-PGJ_2_), which exerts its anti-inflammatory activity through activation of ARE, suppresses carrageenan induced pleurisy [14]. Thus, coordinate induction of cytoprotective genes via activation of AREs may represent a novel therapeutic approach for the treatment of immune and inflammatory diseases [8], [10], [15].

Here we show the cytoprotective effects of BTZO-15, a BTZO-1 derivative with favorable absorption-distribution-metabolism-elimination-toxicity (ADME-tox). BTZO-15 suppressed NO-onduced cell death in IEC-18 cell line derived from rat intestinal epithelial cells. BTZO-15 also showed protective effects against dextran sulfate sodium (DSS)-induced colitis and 2,4,6-trinitrobenzene sulfonic acid (TNBS)-induced colitis in rats. To date, no ARE activator have been used as therapeutic drugs for IBD. The results suggest that ARE activation can be an attractive and novel approach to IBD therapy.

## Results

### BTZO-15 interacts with MIF

**Figure 1:**
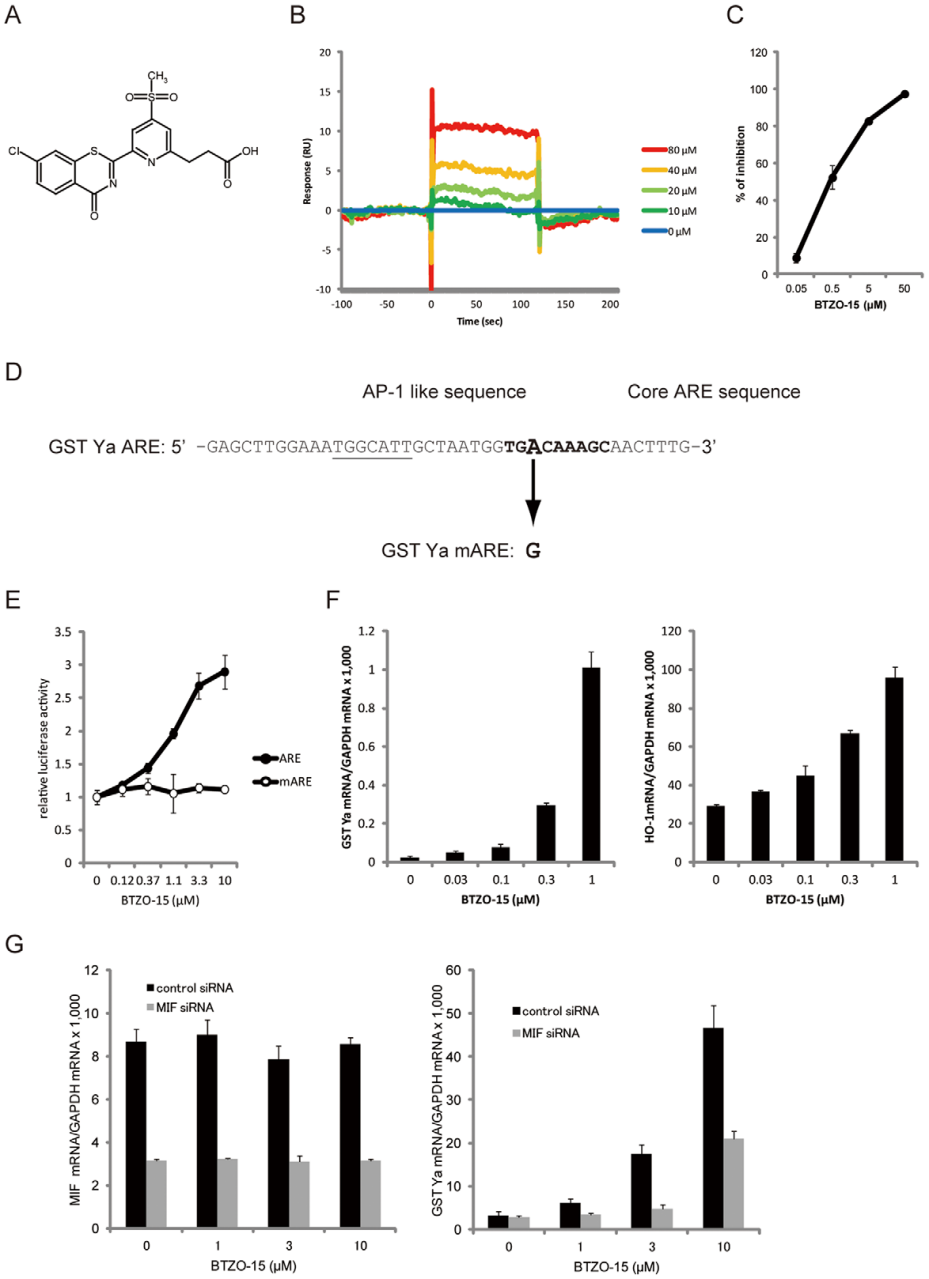
BTZO-15 activates the GST Ya ARE by interacting with MIF. (A) Chemical structures of BTZO-15. (B) Sensorgram showing binding of BTZO-15 to immobilized hMIF on a CM5 sensor chip using an SPR biosensor. (C) Displacement studies with BTZO-15 by the SPA showing inhibition of BTZO-1 binding to captured rMIF on SPA beads. Results shown are the mean ± SD, n = 3. (D) Nucleotide sequence of the rat GST Ya ARE. The core ARE sequence is indicated by the nucleotides in boldface type. (E) Reporter gene assays showing the effect of BTZO-15 on ARE activity. H9c2 cells transiently transfected with either pGL3-ARE-Luc or pGL3-mARE-Luc reporter plasmids were treated with the indicated concentrations of BTZO-15 for 24 h and luciferase activities were measured. Results shown are the mean ± SD, n = 3. (F) BTZO-15 induced ARE-regulated cytoprotective proteins in H9c2 cells. The cells were treated with the indicated concentrations of BTZO-15 for 6 h (HO-1) or 21 h (GST Ya). HO-1 mRNA levels were measured by real-time PCR and normalized by GAPDH mRNA. Results shown are the mean ± SD, n = 3. (G) Effects of reducing the cellular MIF protein level on BTZO-15-induced GST Ya and HO-1 gene expression in H9c2 cells. H9c2 cells transfected with control siRNA (control siRNA) or siRNAs directed against rMIF (MIF siRNA) were treated with 3 µM BTZO-15 with or without 0.8 µM rMIF for 24 h. Messenger RNA levels of MIF, GST Ya, and HO-1 were measured by real-time PCR. MIF protein level in cell lysates was measured by ELISA.

The binding of BTZO-15 (Fig. 1A) to purified recombinant human MIF (hMIF) was analyzed using a surface plasmon resonance (SPR) biosensor. hMIF was immobilized on the active sensor chip CM5 with a surface density of 12,080 resonance units (RU) (MIF sensor chip). Analysis of the BTZO-15 solution revealed dose-dependent binding signals to the MIF sensor chip (Fig. 1B).

Next, binding of BTZO-15 to purified recombinant rat MIF with a 6His-tag to C terminus (rMIF-His) was measured by a scintillation proximity assay (SPA) which measured the competitive inhibition of the radio-labeled [^3^H]-BTZO-1 binding to rMIF-His [8]. BTZO-15 inhibited the binding of [^3^H]-BTZO-1 to rMIF-His in a dose-dependent manner (Fig. 1C). These results, and those of the SPR biosensor analysis, suggested that BTZO-15 directly interacts with MIF.

### BTZO-15 activates ARE-mediated transcriptional induction

The effect of BTZO-15 on ARE activation was investigated using a luciferase reporter assay in H9c2 cells. The pGL3-ARE-Luc reporter plasmid, which expresses luciferase under the control of a rat GST Ya ARE (Fig. 1D), and the pGL3-mARE-Luc reporter plasmid, in which a point mutation (A to G) was introduced into the core sequence (TGACAAAGC) to almost completely abolish its activity, were used in this assay (Fig. 1D) [8], [16]. When H9c2 cells transfected with pGL3-ARE-Luc, but not pGL3-mARE-Luc, were treated with BTZO-15, luciferase activity was induced in a dose dependent manner (Fig. 1E). Thus, increment values of luciferase activity in cells transfected with pGL3-ARE-Luc are ARE-dependent. Accordingly, BTZO-15 induced expression of ARE-regulated genes, such as HO-1 and GST Ya, in H9c2 cells; BTZO-15 increased both HO-1 and GST Ya mRNA expression in a dose dependent manner (Fig. 1F).

### Reduction in cellular MIF levels decreases BTZO-15-induced GST Ya mRNA expression

The effects of reducing the cellular MIF protein level on BTZO-15-induced GST Ya mRNA expression were investigated by lowering the MIF mRNA level with small interfering RNA (siRNA). A reduction of MIF mRNA level was attained by MIF siRNA transfection into H9c2 cells (Fig. 1G). Under these conditions, induction of GST Ya mRNA expression by BTZO-15 was significantly reduced (Fig. 1G).

### BTZO-15 induces HO-1 mRNA levels and suppresses NO-induced cell death in IEC-18 cells

**Figure 2:**
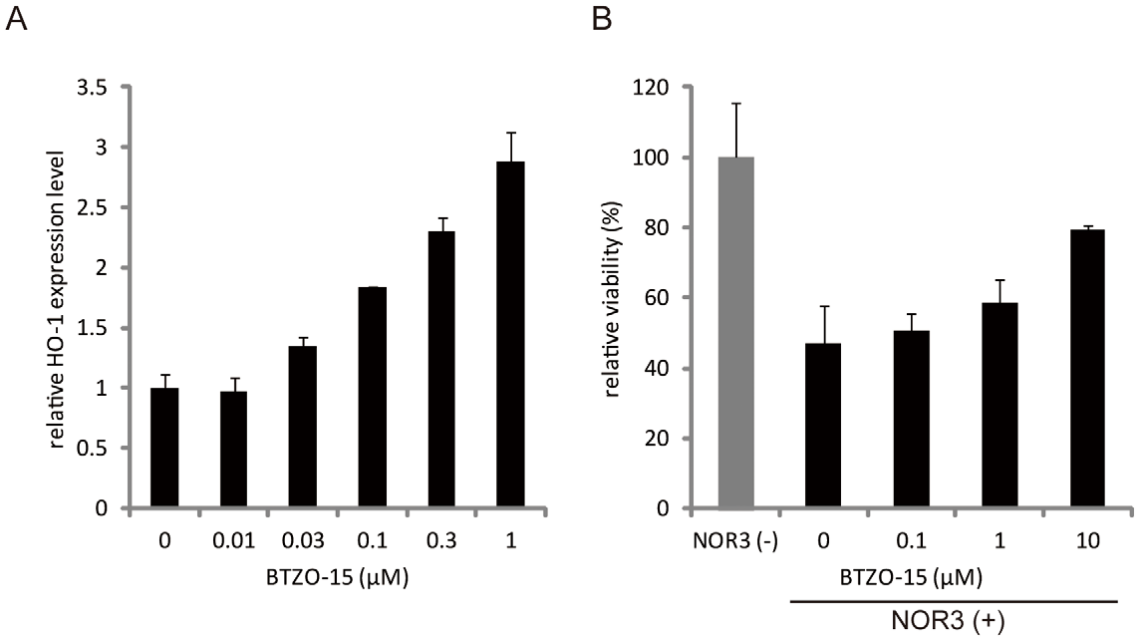
BTZO-15 increases HO-1 mRNA levels and suppresses NO-induced cell death in IEC-18 cells. (A) Real-time PCR expression analysis showing the effects of BTZO-15 on the induction of HO-1 mRNA in IEC-18 cells. The cells were treated with the indicated concentrations of BTZO-15 for 24 h. HO-1 mRNA levels were measured by real-time PCR and normalized by GAPDH mRNA. Results shown are the mean ± SD, n = 3. (B) Cell death inhibitory activity of BTZO-15 in IEC-18 cells. The cells were cultured in the presence of either vehicle or the indicated concentrations of BTZO-15 in serum free medium for 1 h. The cells were then treated with 130 µM NOR3 and cultured for 24 h. Cell viability was determined using the WST-8 cell respiratory assay. The experimental value for cell death inhibitory activities is expressed as relative viability and is the mean ± SD, n = 3.

The effect of BTZO-15 on expression of HO-1 was evaluated using IEC-18 cells. BTZO-15 increased HO-1 mRNA levels in a dose dependent manner (Fig. 2A). GST Ya mRNA could not be measured, probably due to its low level of expression in IEC-18 cells. The effect of BTZO-15 on NO-induced cell death was also examined in IEC-18 cells with Cell Counting Kit-8. Stimulation of IEC-18 cells with 130 µM of NOR3, a NO-generator, reduced cell viability. BTZO-15 treatment suppressed the NO-induced cell death in a dose-dependent manner (Fig. 2B).

### BTZO-15 ameliorates DSS-induced colitis in rats

**Figure 3:**
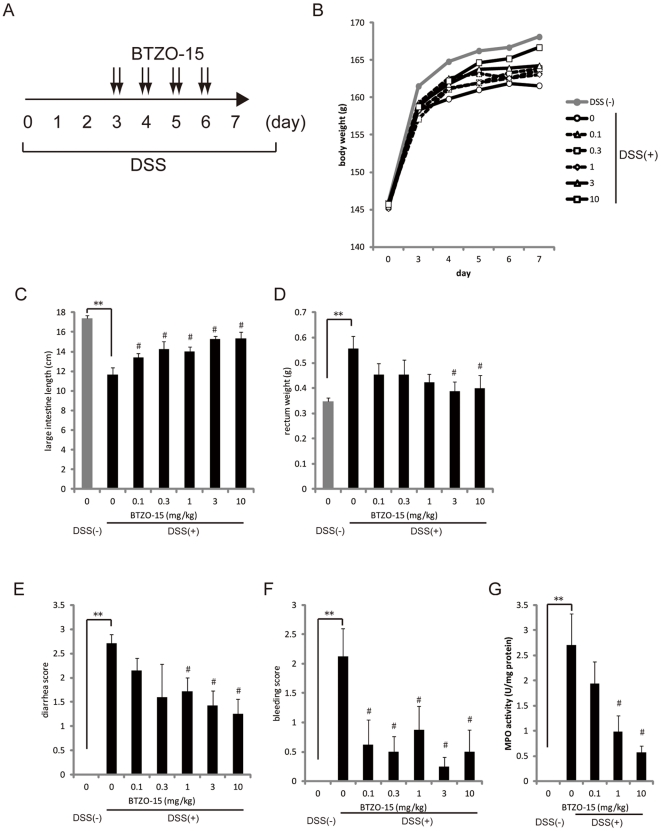
BTZO-15 ameliorates DSS-induced colitis in rats. Colitis was induced in male F344/Du rats by daily treatment with 2.3% DSS solution in drinking water for 7 days. BTZO-15 was orally administered to rats at the indicated doses of 0.1, 0.3, 1, 3, and 10 mg/kg (b.i.d.) for 4 days from experimental day 3 to 6 (A). Body weight (B) was measured every day. At experimental day 7, the rats were sacrificed, and large intestine length (C), rectum weight (D), diarrhea score (E), bleeding score (F), and MPO activity (G) underwent a double blind evaluation. All data except body weight shown are mean ± S.E.M. Body weight data shown are means, n = 8. ** P≤0.01: compared the DSS (−) vehicle control group with DSS (+) vehicle control group using Student's t test, and # P≤0.025: compared the DSS (+) vehicle control group with DSS (+) + BTZO-15 treated groups by one-tailed Williams's test (C and D). **P≤0.01: compared the DSS (−) vehicle control group with DSS (+) vehicle control group using Wilcoxon test, and # P≤0.025: compared the DSS (+) vehicle control group with DSS (+) + BTZO-15 treated groups by one-tailed Shirley-Williams test (E and F). ** P≤0.01: compared the DSS (−) vehicle control group with DSS (+) vehicle control group with Aspin-Welch test, and # P≤0.025: compared the DSS (+) vehicle control group with DSS (+) + BTZO-15 treated groups with one-tailed Williams's test (G).

The effect of BTZO-15 on DSS-induced colitis rats was assessed in a double blinded study; body weight, stool consistency/diarrhea score, bleeding score, large intestine length, and rectum weight were evaluated. The addition of 2.3% DSS to drinking water for 7 days caused colitis with a reduction in body weight and large intestine length, and an increase in the rectum weight, stool consistency/diarrhea score, bleeding score, and rectal MPO activity (Fig. 3A–G). Oral administration of BTZO-15 for 4 days from experimental day 3 to 6 improved all parameters in a dose-dependent manner (Fig. 3B–G).

### BTZO-15 increases HO-1and GST Ya protein levels in the rectum of DSS induced colitis rats

**Figure 4:**
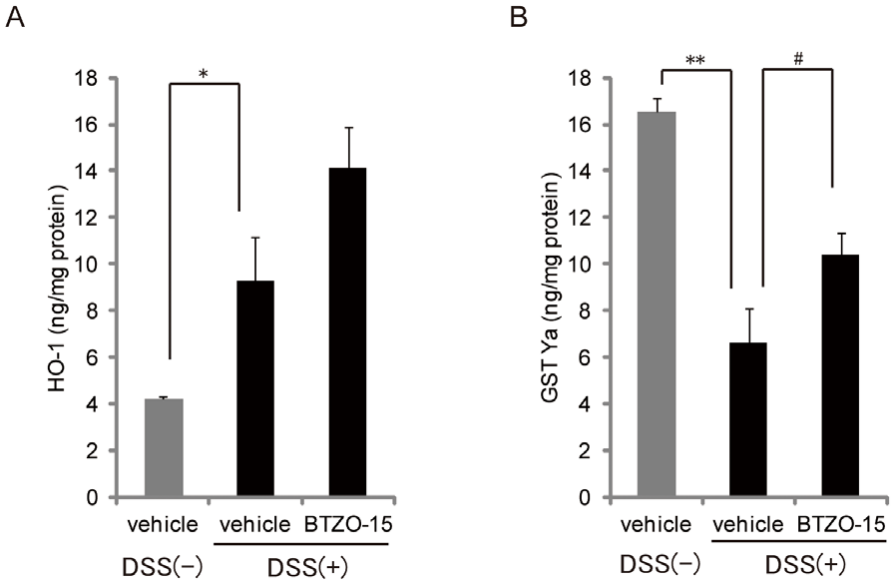
BTZO-15 induces HO-1 and GST Ya protein expression in the rectum of rats with DSS-induced colitis. Colitis was induced in rats by daily treatment with 2.3% DSS solution in drinking water for 7 days. During this period, the rats were orally administered BTZO-15 (3 mg/kg, b.i.d.) or vehicle (0.5% MC). Rectal HO-1 (A) and GST-Ya (B) protein expression was assessed after excision and homogenization in PBS supplemented with 0.1% NP-40. The amount of GST Ya and HO-1 protein was quantified using ELISA. Results shown are the mean ± S.E.M, n = 8. * P≤0.05: compared the DSS (−) vehicle control group with DSS (+) vehicle control group using Aspin-Welch test. # P≤0.05: compared the DSS (+) vehicle control group with DSS (+) + BTZO-15 treated groups with Student's t test.

The effect of BTZO-15 on the expression of rectal HO-1 and GST Ya was studied in rats with DSS-induced colitis. DSS treatment for 7 days resulted in an increase in the level of rectal HO-1 protein (Fig. 4A). Oral administration of BTZO-15 (10 mg/kg) tended to increase HO-1 protein levels (Fig. 4A). Decreased rectal levels of GST Ya protein were significantly recovered by oral administration of BTZO-15 (10 mg/kg) (Fig. 4B).

### BTZO-15 ameliorates TNBS induced colitis in rats

**Figure 5:**
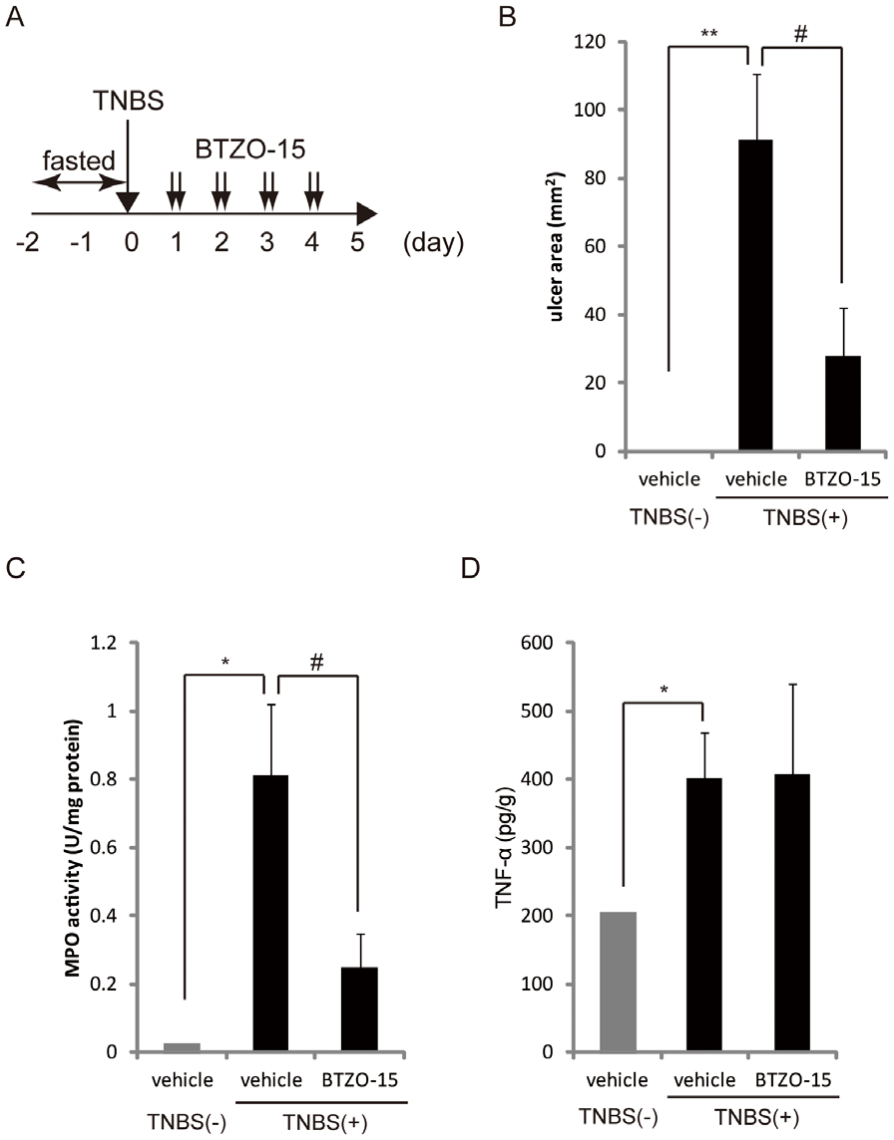
BTZO-15 ameliorates TNBS-induced colitis in rats. Colitis was induced in male F344/Du rats by topical intracolonic application of a TNBS solution. BTZO-15 was orally administered to rats at the indicated doses of 10 mg/kg (b.i.d.) for 4 days from experimental day 1 to 4 (A). The efficacy of BTZO-15 was evaluated using the size of the rectal ulcer (B), MPO activity (C), and TNF-α level in rectum. Results shown are the mean ± S.E.M. TNBS-treated group, n = 6; TNBS untreated group, n = 2. ** P≤0.01: compared the DSS (−) vehicle control group with DSS (+) vehicle control group by Aspin-Welch test, and # P≤0.05 compared the DSS (+) vehicle control group with DSS (+) + BTZO-15 treated groups with Student's t test (B). * P≤0.05: compared the DSS (−) vehicle control group with DSS (+) vehicle control group using Aspin-Welch test, and # P≤0.05 compared the DSS (+) vehicle control group with DSS (+) + BTZO-15 treated groups by Aspin-Welch test (C and D).

We next evaluated the effects of BTZO-15 on TNBS-induced colitis in rats (Fig. 5A). TNBS administration led to ulcers in the rectum, increased rectal MPO activity, and up-regulated TNF-α levels (Fig. 5B–D). Oral administration of BTZO-15 significantly decreased the ulcerated area and rectal MPO activity (Fig. 5B, C) without affecting rectal TNF-α levels (Fig. 5D).

## Discussion

The etiology of IBD is still unclear, although there is evidence that oxidative stress plays a role in the pathogenesis of IBD and could be the mainstay of disease initiation and perpetuation [3]–[5]. Many agents, such as 5-amino salicylic acid (5-ASA), antibiotics, steroids, immunosuppressive agents, and anti tumor necrosis factor (TNF)-α, infliximab, are available for IBD therapy; however, they have problems of lower efficacy and/or side effects. For example 5-ASA, a pharmacological standard therapy for UC, [2], [17], does not exhibit adequate efficacy in maintenance and remission and produces side-effects, such as interstitial nephritis [2], [17]. Antibiotics, steroids, and immunosuppressive agents, including cyclosporine, are powerful therapeutic approaches to avoid sepsis or life-threatening complications in IBD in an acute setting; however, their routine use has been hindered by side-effects that include infectious complications [2], [17]. Although infliximab has attracted attention for its curative effect on moderate/severe UC and CD, there are problems of resistance and side effects upon long term dosage [2], [17], [18]. Thus, novel therapeutic drugs with better efficacy and side effects profiles are needed.

Both DSS- and TNBS-induced colitis are considered useful experimental models for IBD [19]. DSS is toxic to colonic epithelial cells in the basal crypts, and elicits an inflammatory response that causes colitis similar to human UC [19], [20]. BTZO-15 significantly suppressed the typical symptoms of DSS-induced colitis in rats: large intestine shortening, rectum weight gain, diarrhea, and bleeding. BTZO-15 also suppressed an increase in rectal MPO activity, which is an index of tissue-associated neutrophil accumulation. In contrast, TNBS-induced colitis is believed to induce a T-cell-mediated response against hapten-modified autologous proteins/luminal antigens similar to a Th1 immune response and comparable to the inflammatory processes present in human CD [21], [22]. BTZO-15 significantly decreased the ulcer-affected area with suppression of rectal MPO activity in rats with TNBS-induced colitis. Interestingly, BTZO-15 did not affect the rectal TNF-α level. These results indicate that BTZO-15 has potential as a novel therapeutic drug for both UC and CD.

BTZO-15 induced expression of HO-1 and suppressed NO-induced cell death in a rat intestinal epithelial cell line (IEC-18). Reactive oxygen species (ROS), such as NO, play an important role in the pathogenesis of various inflammatory diseases, including IBD [4]–[6], and ARE is an important countermeasure against oxidative stress in mammalian cells [10]–[12]. Thus, ARE activation could be an attractive strategy for IBD therapy. Here we show that DSS treatment increased HO-1 protein levels in the rectum and BTZO-15 further increased HO-1 protein levels. This HO-1 up-regulation by DSS may be an intrinsic defense mechanism against oxidative stress [23]. GST-Ya levels in the rectum decreased by DSS treatment. This GST-Ya down-regulation by DSS is in accord with a previous report that GSTs levels and GSTs activities are reduced by DSS treatment [24], [25]. BTZO-15 significantly recovered GST-Ya levels in the rectum. These results are in accord with a previous report that GSTs levels and GSTs activities are reduced by DSS treatment [24], [25]. Serum TNF-α was up-regulated in rats with TNBS-induced colitis, and BTZO-15 did not affect TNF-α levels. These results, and those of the *in vitro* experiments, suggest that the therapeutic effects of BTZO-15 in animal models of IBD are based on increases in HO-1 and GST Ya levels via ARE activation in rectum, not suppression of cytokine production, although further studies will be needed to clarify the precise mechanisms of action.

It is evident that activation of ARE-regulated genes contributes to the regulation of cellular antioxidant defense systems, and pharmacological activation of endogenous cytoprotective proteins through ARE activation is predicted to serve as a novel strategy for the treatment of cardiovascular and inflammatory diseases [10], [15]. Thus, BTZO-1 derivatives appear to have therapeutic potential for a broad range of diseases caused by oxidative stress. In fact, BTZO-2, an active BTZO-1 derivative of ARE activation, confers protection to heart tissues during ischemia/reperfusion injury in rats [8]. Evaluation of the effects of BTZO-1 derivatives on other oxidative stress-related disease models is worthy of additional investigation.

A forward chemical genetics approach has been used to show that BTZO-1 derivatives may activate AREs via binding to MIF [8], [26]. Here we show that BTZO-15 directly interacts with MIF and induces ARE-mediated expression of cytoprotective genes, such as GST-Ya and HO-1. This induction of GST Ya mRNA expression by BTZO-15 was decreased in MIF siRNA-transfected H9c2 cells. These results, and those of our previous report [8], suggest that BTZO-15 activates AREs by binding to MIF. MIF reportedly contributes to the symptoms of IBD, and inhibition of MIF could be a potential treatment [27]–[29]. However, our results suggest that activation of MIF by BTZO-15 induces ARE activation, leading to the therapeutic effects in IBD models. Further studies are needed to reveal how MIF participates in IBD and how BTZO-15 works in this system.

In summary, we demonstrate that BTZO-15 activates ARE-mediated gene expression and suppresses NO-induced cell death *in vitro*. In addition, BTZO-15 ameliorates both DSS- and TNBS-induced colitis in rats. Since no ARE activator have been used as therapeutic drugs for IBD, BTZO-15 could be a novel drug for IBD with potent therapeutic activity and a low side effects profile. Our studies further demonstrate that ARE activation can be an attractive and novel approach to IBD therapy.

## Materials and Methods

### Experimental animals

#### Ethics statement

The care and use of the animals and experimental protocol were approved by the Experimental Animal Care and Use Committee at Takeda Pharmaceutical Company (Approval ID: TEACUC-1822, TEACUC-1824, and TEACUC-2414).

Seven-week-old male F344/Du rats were obtained from Charles River Laboratories Japan Inc. (Yokohama). The rats were housed 4 per cage in specific pathogen-free and standard controlled environmental conditions with a 12-h light/dark cycle and with food (MF; Clea Japan Inc., Tokyo) and water provided ad libitum.

### Materials

BTZO-15 was synthesized at Takeda Pharmaceutical Company Limited. Detailed methods for the synthesis of BTZO-15 will be described elsewhere. Recombinant human MIF-His (hMIF) and recombinant rat MIF (rMIF) were prepared as described previously [8].

### Surface plasmon resonance (SPR) analysis

The interaction of MIF with BTZO-15 was examined using the BIACORE 3000 System (GE Healthcare UK Limited) at 25°C. Human MIF (hMIF) was immobilized with a surface density of 12,080 resonance units (RU) on an activated sensor chip CM5 according to the manufacturer's instructions. A reference surface was prepared without hMIF in flow cell 1. The interaction of BTZO-15 with hMIF was tested in PBS containing 3% DMSO under a 30 µM/min continuous flow at 25°C. Sixty microliters of sample containing the indicated concentrations of compounds were injected onto the sensor chip. The sensor surfaces were regenerated to remove all bound analyte between binding cycles by serial injections of 5 µL of 10 mM NaOH at a flow rate of 60 µL/min. Binding data were analyzed using the BIACORE 3000 control software and BIAevaluation Software (GE Healthcare UK Limited).

### Scintillation proximity assay (SPA)

The [^3^H]-BTZO-1/rMIF-His SPA assay was conducted as described previously [8]. In the SPA measuring the competitive inhibition of [^3^H]-BTZO-1 binding to rMIF-His, 62.5 µg of Ysi (2–5 µm) copper His-tag SPA beads and 0.25 µg of rMIF-His protein were incubated in PBS containing 0.025% Triton X-100 at 4°C overnight. Subsequently, the indicated concentrations of BTZO-15 and 20 nM of [^3^H]-BTZO-1 were added to each well to give a final volume of 100 µL. After a 3-h-incubation, radioactivity derived from the bound [^3^H]-BTZO-1 was measured using a microplate scintillation counter. The final concentration of solvent was kept to 0.5% DMF.

### ARE-Luc reporter assays

The ARE-Luc reporter assay used the reporter plasmids pGL3-ARE-Luc and pGL3-mARE-Luc [8]. In brief, H9c2 (ATCC, Manassas, VA, USA) cells were cultured in D-MEM supplemented with 10% FBS and 1% penicillin and streptomycin (PS) for about 16 h before they were transfected with pGL3-ARE-Luc or pGL3-mARE-Luc using FuGENE6 transfection reagent (Roche, Indianapolis, IN, USA) according to the manufacturer's instructions. After a 7-h-incubation, the cells were harvested in a trypsin-EDTA solution, suspended in D-MEM supplemented with 10% FBS and 1% PS, and cultured in 96-well plates (Becton Dickinson, San Jose, CA, USA) for 17 h at a density of 1×10^4^ cells/well. The cells were washed three times with DMEM supplemented with 1% PS and further cultured with various concentrations of BTZO-15 for about 24 h. Cell lysates were prepared using the Steady-Glo Luciferase Assay System (Promega, Madison WI, USA) and luciferase activity was measured with the WALLAC ARVO SX (GE Healthcare UK Limited, Buckinghamshire, UK). All samples were dissolved in DMF, with the final concentration of DMF at 0.1%.

### Real-time PCR analysis of HO-1 and GST Ya mRNA expression in H9c2 cells

H9c2 cells were suspended in DMEM including 10% FBS, 2 mM glutamine, and 1% PS at a density of 3.0×10^4^ cells/well and cultured in 12-well plates at 37°C in 5% CO2/95% humidified air for 1 day. The cell were washed three times with DMEM supplemented with 2 mM glutamine and 1% PS. After medium change with DMEM supplemented with 2 mM glutamine and 1% PS, the indicated concentrations of BTZO-15 were added to the culture medium and incubated for 6 h or 21 h for expression analysis of HO-1 or GST Ya, respectively. For quantitative PCR, total RNA was extracted with RNeasy 96 Kit (QIAGEN GmbH, Hilden, Germany) following the manufacturer's instructions and further purified by DNase digestion using MessageClean (GenHunter Corporation, Nashville, TN, USA). Real-time PCR was carried out with an ABI PRISM 7900HT sequence detection system (Life Technologies, Carlsbad, CA, USA) using TaqMan reagents (Life Technologies). RNA was normalized using glyceraldehyde-3-phosphate dehydrogenase (GAPDH) TaqMan probes according to the manufacturer's instructions. The following primers and probe were used for HO-1 analysis: forward primer (rHO-1F2), 5′-CCGCCTTCCTGCTCAACA-3′; reverse primer (rHO-1R2), 5′-AAGAAACTCTGTCTGTGAGGGACTCT-3′; TaqMan probe (rHO-1-2), 5′-FAM-CAGGCACTGCTGACAGAGGAACACAAAG-TAMRA-3′. The FAM (6-carboxyfluorescein) reporter is covalently linked to the 5′ end of the probe; TAMRA (6-carboxy-N, N, N′, N′-tetramethyl-rhodamine), located at the 3′ end of the probe, is used for quenching. The following primers and probes were used for GST Ya analysis: forward primer (rGSTYaF1), 5′-TGCCAGCCTTCTGACCTCTTT-3′; reverse primer (rGSTYaR1), 5′-CTGCAGGAACTTCTTCACATTGG-3′; TaqMan probe (rGSTYa-1), 5′-FAM-AAGGCCTTCAAGAGCAGAATCAGCAGC-TAMRA-3′. The following primers and probe were used for GAPDH analysis: forward primer (rGAPDHF2), 5′-TGCCAAGTATGATGACATCAAGAAG-3′; reverse primer (rGAPDHR2), 5′-AGCCCAGGATGCCCTTTAGT-3′; TaqMan probe (rGAPDH-2), 5′-VIC-TGGTGAAGCAGGCGGCCGAG-TAMRA-3′.

### RNA interference

RNA interference was carried out as described previously [8]. H9c2 cells were suspended in DMEM supplemented with 10% FBS and 1% PS at a density of 1×10^5^ cells/mL and cultured for 1 day. After washing three times with DMEM supplemented with 3% FBS, the cells were transfected with MIF siRNA (5′-GCUCAUGACUUUUAGUGGC-3′) (Life Technologies) or control siRNA (4611, Life Technologies) (100 nM), using Opti-MEM (Life Technologies) and Oligofectamine (Life Technologies), and cultured for 1 day. The cells were then washed three times with DMEM supplemented with 3% FBS and cultured for 3 days. The cells were harvested with a trypsin-EDTA solution, suspended in DMEM supplemented with 3% FBS at a density of 5×10^4^ cells/mL and cultured for 1 day. The cells were again transfected with either MIF siRNA or control siRNA (100 nM) using Opti-MEM and Oligofectamine, cultured for 2 days, washed three times with DMEM, and then incubated with the indicated concentrations of BTZO-15 for 24 h. Total RNA was extracted using an RNeasy 96 Kit (QIAGEN GmbH) following the manufacturer's instructions.

### Real-time PCR analysis of HO-1 and GST Ya mRNA expression in IEC-18 cells

IEC-18 cells were suspended in MEM including 10% FBS and 1% PS at a density of 1.5×10^4^ cells/well and cultured in 48-well plates at 37°C in 5% CO2/95% humidified air for 1 day. After a medium change with MEM supplemented with 1% PS, the cells were pretreated with various concentrations of BTZO-15 for 7.5 h. Quantitative PCR was carried out as described above.

### Cell survival assays for the evaluation of NO-induced cell death

IEC-18 cells were suspended in MEM supplemented with 10% FBS and 1% PS at a density of 1.5×10^4^ cells/well and cultured in 96-well plates at 37°C in 5% CO_2_/95% humidified air for 1 day. After a medium change with MEM plus 1% PS, cells were pretreated with various concentrations of BTZO-15 for 1 h. Cell death was induced by treatment with 130 µM of (±)-(E)-4-Ethyl-2-[(E)-hydroxyimino]-5-nitro-3-hexenamide (NOR3; Dojindo, Makishi), which generates nitric oxide (NO), for 1 day. Afterwards, cell viability was evaluated with the Cell Counting Kit-8 (Dojindo).

### Induction and treatment of DSS-induced colitis

F344 DuCrj rats, except the normal group, received drinking water with 2.3% (wt/vol.) DSS (molecular weight 5,000 Da; Wako Pure Chemical Industries Co., Ltd., Osaka) for 7 days, from experimental day 0 (starting day) to experimental day 7. Control rats received drinking water without DSS.

BTZO-15 was suspended in a 0.5% methylcellulose solution and administered orally twice per day at an interval of 4 h in a volume of 5 mL/kg from experimental day 3 to day 6 (n = 8 rats in each group). The rats were sacrificed under ether anesthesia by decapitation. The efficacy of BTZO-15 in this model was evaluated in a double-blind fashion using the following parameters; body weight, stool consistency/diarrhea score (0 points = normal and formed, 1 points = pasty and formed, 2 points = pasty to soft and unformed, 3 points = diarrhea), bleeding score (0 points = no bleeding, 1 points = slight bleeding, 2 points = obvious bleeding, 3 points = mostly bleeding) [30], large intestine length from appendix subjacency to anus, rectum weight from anus to 5 cm, and myeloperoxidase (MPO) activity.

### MPO activity

MPO activity was measured to monitor the degree of neutrophil infiltration in the rectum. Rats were sacrificed under ether anesthesia by decapitation and rectums were removed and gently cleared of feces and rinsed with chilled PBS. The specimens were weighed, cut into small pieces in 0.5% hexadecyl trimethyl-ammonium bromide (HTAB) (Sigma-Aldrich, St. Louis, MO, USA)/50 mM KH_2_PO_4_ and Na_2_PO_4_ buffer (pH 6.0), and homogenized with a Physcotron (Microtec Co., Ltd., Funabashi). The homogenates were subjected to two sonication and freeze-thaw cycles. The suspensions were centrifuged at 3,000 rpm for 10 min at 4°C and the supernatants were reacted with a Peroxidase Colorimetric Kit (Sumitomo Bakelite Company Limited, Tokyo). Myeloperoxidase Polymorphonuclear Leukocytes (Merck KGaA, Darmstadt, Germany) were used as a standard and the data were presented as units per milligram of protein.

### Rat GST-Ya ELISA

The rat GST Ya protein was prepared using a pET system (Merck KGaA) as described previously [8]. Anti-GST serum was generated by immunizing SPF Japanese white rabbits with recombinant GST Ya protein (MBL, Nagoya). Anti-GST Ya IgG was purified from the serum using protein-G affinity chromatography (GE Healthcare UK Limited) following the manufacturer's instructions. Rectal concentrations of GST Ya were quantified with a sandwich ELISA using polyclonal rabbit anti-GST Ya detector, a biotin-labeled polyclonal rabbit anti-GST Ys detector, and purified recombinant rat GST Ys protein.

### Analysis of rectal GST-Ya and HO-1 protein levels

Rats were anesthetized with ether and sacrificed by decapitation. Rectums were quickly removed and gently cleared of feces and rinsed with chilled PBS. The specimens were excised in 4 volumes of PBS supplemented with nonidet P-40 (NP-40) and homogenized with a Teflon homogenizer. The homogenates were centrifuged at 1,000× g for 5 min and the supernatants were retained. Protein content was determined with a BCA Protein Assay Kit (Thermo Fisher Scientific Inc., Rockford, IL, USA) and equal protein levels of the supernatants were used to evaluate the concentrations of GST-Ya and HO-1 using rat GST-Ya ELISA and rat Heme Oxygenase-1 EIA Kit (Takara Bio Inc., Ohtsu), respectively.

### Induction and assessment of TNBS-induced colitis

Seven-week-old male F344/Du rats were given water but no food for 48 h. After fasting, at experimental day 0, the rats were anesthetized with 30 mg/kg pentobarbital, and colitis was induced by topical intracolonic application of 0.4 mL of a solution containing 4 mg of TNBS (Wako Pure Chemical Industries Co., Ltd.) in 50% ethanol (Wako Pure Chemical Industries Co., Ltd.) with a rubber cannula. The rats received 10 mg/kg of BTZO-15 orally twice per day at an interval of 4 h from experimental day 1 to day 4 (n = 8 rats in each group). On experimental day 5, the rats were sacrificed under ether anesthesia by decapitation and their rectums were dissected. The efficacy of the BTZO-15 in this model was evaluated based on the size of the ulcerated area on the rectum, which was quantified by MacScope, MPO activity, and TNFα contents.

### Statistical analysis

In Figure 3, the statistical significance of the difference between the vehicle- and DSS-treated groups on diarrhea and bleeding scores were determined by Wilcoxon's test. Statistical significance between two groups was also determined using the Student's t test for homogenous data or Aspin-Welch test for non-homogenous data. In the experiments that examine the effect of multiple doses of test compounds, the statistical significance was determined by a one-tailed Williams test for homogenous data or a one-tailed Shirley-Williams test for non-homogenous data.
